# Iron Chelation Therapy With Deferasirox in Sickle Cell Disease With End-Stage Renal Disease

**DOI:** 10.7759/cureus.24146

**Published:** 2022-04-14

**Authors:** Ashok Raj, Kerry McGowan, Esther Knapp, Jun Zhao, Siddharth Shah

**Affiliations:** 1 Pediatric Hematology and Oncology, University of Louisville, Louisville, USA; 2 Pediatric Nephrology, University of Louisville, Louisville, USA

**Keywords:** hemodialysis, sickle cell disease, end-stage renal disease, anca, deferasirox

## Abstract

Patients with transfusion‐dependent sickle cell disease (SCD) are at risk of iron overload and its complications. Iron overload is a significant risk factor for chronic liver disease in patients who are dependent on hemodialysis secondary to end-stage renal disease (ESRD). Deferasirox is being increasingly used as an iron-chelating agent for the treatment of iron overload in both adults and children. There are limited reports on its use in pediatric patients with ESRD. Here, we discuss the use of deferasirox to treat iron overload in a 15-year-old male with SCD, ESRD from granulomatosis with polyangiitis, and dependent on hemodialysis. We also review the literature on similar uses of deferasirox in adult patients with ESRD.

## Introduction

Patients with transfusion‐dependent sickle cell disease (SCD) are at risk of iron overload and its clinical complications [1]. Iron overload is a significant risk factor for chronic liver disease in patients who are dependent on hemodialysis secondary to end-stage renal disease (ESRD) [2]. Deferasirox is being increasingly used as an iron-chelating agent for the treatment of iron overload in both adults and children. There are limited case reports on the use of deferasirox in ESRD patients, including transfusion-dependent beta-thalassemia major patients, an SCD patient on peritoneal dialysis, and chronic kidney disease (CKD) due to other causes [3-5].

ESRD is often associated with anemia because of decreased erythropoietin production, iron absorption, and iron bioavailability. In the ESRD setting, the increased production of hepcidin decreases iron bioavailability [6]. Serum ferritin is extensively used to measure iron loading from chronic transfusion because it is standardized, economical, and readily accessible [7]. But data from national STOP (Stroke Prevention Trial in Sickle Cell Anemia) trials suggest that the correlation between serum ferritin and liver iron concentration (LIC) is not optimal [8]. Serum ferritin can be affected by factors such as inflammation and infection and has also been shown to be inadequate for assessing iron status in subjects with CKD. In addition, 60% of hemodialysis patients have a LIC that is greater than twice the upper limit of normal [9]. There is limited information on effective iron-chelating agents in pediatric patients with SCD and ESRD. Here, we report the effective use of deferasirox in a child with SCD with ESRD.

## Case presentation

Our patient was a 15-year-old male with Hb SCD, diagnosed on his newborn screen. He had not had any significant sickle cell-related complications until he was diagnosed with antineutrophil cytoplasmic antibodies (c-ANCA)-associated vasculitis. He initially presented at an outside facility with right middle lobe (RML) pneumonia associated with effusion. Subsequently, he had several events, including splenic sequestration, acute renal failure requiring dialysis, an episode of posterior reversible encephalopathy syndrome (PRES), infarct to the left occipital lobe, pulmonary hemorrhage, and cavitary pulmonary lesions. Initially, the acute kidney injury was related to acute tubular necrosis (ATN), but three months after initial presentation, he was found to have high titers of proteinase 3-ANCA antibodies. A renal biopsy was performed at that time that showed pauci-immune sclerosing glomerulonephritis associated with 100% glomerulosclerosis. He was subsequently diagnosed with granulomatosis with polyangiitis (GPA) and started on induction therapy with cyclophosphamide, methylprednisolone, and plasmapheresis. A month later, he was re-presented to the hospital with seizures, altered mental status, temporary blindness, and found to have the evolution of previous left occipital lobe infarct. He also had new multifocal regions of restricted cortical diffusion compatible with tiny infarcts and resolution of PRES. Due to concerns that these infarcts were secondary to his vasculitis, he was started on a course of rituximab and received four doses.

Because of being hemodialysis dependent for more than three months, he was diagnosed with end-stage renal disease. The hemodialysis procedure was done using Optiflux F160NR dialyzer (with an advanced polysulfone membrane, surface area 1.5 m^2^, Kuf 45 mL/hr/mm Hg, and KoA urea 1064; Fresenius Renal Technologies, Waltham, MA). The blood flow rate during the hemodialysis prescription was 350 mL/min, and the dialysate flow rate was 600 mL/min.

He demonstrated worsening anemia secondary to ESRD, and there was difficulty maintaining hemoglobin at 8-9 g/dL and sickle hemoglobin <30%. He was started on erythrocytapheresis to reduce the risk of sickle cell-related complications. He was placed on erythropoietin to improve hemoglobin production to perform erythrocytapheresis. Due to inconsistent erythropoiesis, he was initially maintained on a combination of erythrocytapheresis and simple transfusion to maintain the above-mentioned parameters. Erythropoietin was administered with each dialysis. Initially, he was on 10,000 units three times a week (TIW). Still, due to a concern that this dose of erythropoietin was worsening his hypertension, the amount was subsequently reduced to 4000 units with dialysis, which was performed three times per week. After one year from the diagnosis of c-ANCA vasculitis, he was maintained only on long-term erythrocytapheresis.

On transfusion therapy, his serum ferritin progressively increased and peaked at 5500 ng/mL after two and a half years of treatment. At that time, he also had an exacerbation of c-ANCA vasculitis with pulmonary and sinus disease. He was initially started on low-dose deferasirox at 5 mg/kg/day due to the family’s concerns about toxicity, but serum ferritin remained in the 3000 ng/mL range. His initial LIC on R2 magnetic resonance imaging, six months from the diagnosis of c-ANCA vasculitis, was 2.7 mg/gm of dry tissue and peaked at 9.4 mg/gm of dry tissue. His dose of deferasirox based on ferritin levels and LIC was gradually increased to 12 mg/kg/day. On a repeat LIC measurement five months after the initiation of the 12 mg/kg/day dose of deferasirox, his liver iron had decreased to 2.6 mg/gm of dry tissue.

At this time, his c-ANCA vasculitis appears to be in remission, and he is off of immunosuppressive therapy. He has ongoing issues with hypertension that is medically managed. He is being considered for haploidentical stem cell transplantation followed by renal transplantation.

## Discussion

The target organ complications in patients with SCD and a significant iron overload appear to be less than those seen in transfusion-dependent beta-thalassemia major patients. However, an increased incidence of iron overload cases resulting in target organ damage, such as liver, is seen in SCD patients after their third decade of life. The iron-related organ damage in SCD is under-recognized among providers [1]. The current clinical practice is to treat iron overload in patients with SCD with the goal of reducing plasma and tissue iron levels to the normal range. The iron load in the body should be kept at low and safe levels. Patient adherence is critical to the success of chelation therapy.

While differences exist in the pathophysiology of iron overload between SCD and beta-thalassemia major or diseases causing bone marrow failure, the treatment is not significantly different. The incidence of iron overload-associated cardiomyopathy is lower in patients with SCD [7].

The predominant excretion route of deferasirox and its metabolites is fecal (approximately 84% ), but at the same time, the renal excretion is very small (8% ) [3]. However, ESRD can significantly affect the pharmacokinetics of deferasirox. Maker et al. showed that when deferasirox was used at a dose of 15 mg/kg compared to 10 mg/kg in their hemodialysis patient treatment groups, the mean plasma concentration of deferasirox increased by 10-fold. The presence of uremia in ESRD patients maintained on hemodialysis patients can lead to decreased fecal excretion or may promote increased intestinal absorption that can result in higher than predicted deferasirox levels at high treatment doses [5].

There are different reports on deferasirox used to treat iron overload in adult patients with ESRD. Yusuf et al. reported the use of deferasirox for six months in a 43-year-old female patient with ESRD secondary to sickle cell nephropathy and managed on peritoneal dialysis. The dose of deferasirox in this patient was 20 mg/kg/day. The treatment with deferasirox helped improve serum ferritin levels, but the treatment was stopped because of symptomatic hypocalcemia that did not respond to increasing dose of calcitriol, calcium supplementation, or increasing the calcium ion concentration in the dialysate solution [4]. To our knowledge, this was one of the initial reports of symptomatic hypocalcemia associated with the use of deferasirox. Tsai et al. reported the use of deferasirox at a dose range of 15-30 mg/kg/day in a 59-year-old male patient with paroxysmal nocturnal hemoglobinuria and maintained on hemodialysis. The improvement in the serum ferritin level was achieved without significant complications in this patient [10]. Hiraga et al. also reported successfully treating iron overload using deferasirox at a dose of 20 mg/kg/day in a 49-year-old male patient with aplastic anemia and ESRD who was receiving hemodialysis [11]. Chen et al. reported another effective use of deferasirox in eight adult hemodialysis patients (age range: 33.6-74.3 years) at a dose of 15 mg/kg/day to treat iron overload secondary to transfusion-dependent anemia of renal disease. The common complications observed were nausea, vomiting, diarrhea, and abdominal pain but there were no serious adverse effects reported [9].

Deferasirox was effectively used in our pediatric patient with no reported adverse effects. Our patient demonstrated a therapeutic response as evidenced by a marked decline in the LIC that was observed at a lower dose level of 12 mg/kg/day. The response was also seen in a short period of time of five months.

## Conclusions

Based on the previously mentioned case series and reports and our patient course, deferasirox can be considered a safe and effective option for SCD patients with ESRD and iron overload given the lack of alternative agents. We recommend monitoring patients for bone marrow, hepatic, gastrointestinal, and other toxicities.
